# Augmented reality-guided periacetabular osteotomy—proof of concept

**DOI:** 10.1186/s13018-020-02066-x

**Published:** 2020-11-17

**Authors:** Pascal Kiarostami, Cyrill Dennler, Simon Roner, Reto Sutter, Philipp Fürnstahl, Mazda Farshad, Stefan Rahm, Patrick O. Zingg

**Affiliations:** 1 Department of Orthopaedics, Balgrist University Hospital, University of Zürich, Forchstrasse 340, 8008 Zürich, Switzerland; 2 Department of Radiology, Balgrist University Hospital, University of Zürich, Forchstrasse 340, 8008 Zürich, Switzerland; 3 Computer Assisted Research & Development Group, Balgrist University Hospital, University of Zürich, Forchstrasse 340, 8008 Zürich, Switzerland

## Abstract

**Background:**

The Ganz’ periacetabular osteotomy (PAO) consists of four technically challenging osteotomies (OT), namely, supraacetabular (saOT), pubic (pOT), ischial (iOT), and retroacetabular OT (raOT).

**Purpose:**

We performed a proof of concept study to test (1) the feasibility of augmented reality (AR) guidance for PAO, (2) precision of the OTs guided by AR compared to the freehand technique performed by an experienced PAO surgeon, and (3) the effect of AR on performance depending on experience.

**Methods:**

A 3D preoperative plan of a PAO was created from segmented computed tomography (CT) data of an anatomic plastic pelvis model (PPM). The plan was then embedded in a software application for an AR head-mounted device. Soft tissue coverage was imitated using foam rubber. The 3D plan was then registered onto the PPM using an anatomical landmark registration. Two surgeons (one experienced and one novice PAO surgeon) each performed 15 freehand (FH) and 15 AR-guided PAOs. The starting point distances and angulation between the planned and executed OT planes for the FH and the AR-guided PAOs were compared in post-intervention CTs.

**Results:**

AR guidance did not affect the performance of the expert surgeon in terms of the mean differences between the planned and executed starting points, but the raOT angle was more accurate as compared to FH PAO (*p* = 0.0027). AR guidance increased the accuracy of the performance of the novice surgeon for iOT (*p* = 0.03). An intraarticular osteotomy performed by the novice surgeon with the FH technique could be observed only once.

**Conclusion:**

AR guidance of osteotomies for PAOs is feasible and seems to increase accuracy. The effect is more accentuated for less-experienced surgeons.

**Clinical relevance:**

This is the first proof of concept study documenting the feasibility of AR guidance for PAO. Based on these findings, further studies are essential for elaborating on the potential merits of AR guidance to increase the accuracy of complex surgical procedures.

## Introduction

Periacetabular osteotomy (PAO) is a well-established procedure for the treatment of hip dysplasia [1, 2] and femoroacetabular impingement caused by true acetabular retroversion [3]. The goal of a PAO is to reorient the acetabulum in a more correct physiological position. Obtaining an ideal acetabular correction is challenging [4] and requires the precise execution of several osteotomies. Furthermore, the osteotomy planes must keep the posterior column intact and also remain extra-articular [5, 6].

Traditional PAO techniques are based on orientation on the bony anatomy alone with or without the addition of intraoperative fluoroscopy as help in orientation to execute and verify the accuracy of the osteotomies. However, fluoroscopy may add to the overall surgical time, as well as increase the patient’s and operating room personnel’s exposure to radiation. Furthermore, it is not sufficient for the control of certain osteotomies due to its two-dimensional nature. Therefore, more sophisticated techniques for the navigation of PAO have been suggested and investigated, including optical tracking systems [7–10] and planar marking and tracking with an inertial measurement unit (IMU) combined into a small hybrid navigation system for PAO [11] as well as patient-specific template guides [12]. Such techniques, however, require additional expensive operating room equipment and/or more extensive surgical exposures. Further, the guidance for the most challenging osteotomy, namely, the ischial osteotomy, cannot be addressed with the currently proposed navigation techniques.

Therefore, there is a need for a simple and radiation-free system to safely perform all the four types of 3D planned osteotomies with greater accuracy. Augmented reality (AR) is an evolving technology with the potential to overcome this challenge. AR can be described as the real-time integration of computer-generated information in the user’s environment. In medicine specifically, AR technology is capable of superimposing a preoperative plan for the user to view. This concept has already aroused interest in the field of medicine since the 1990s [13], but the intraoperative application was not possible until 2012 [14]. Recent technological improvements, namely, optical see-through head-mounted displays (HMD), such as Microsoft’s HoloLens (Microsoft Corporation, Redmond, WA, USA), are more precise and cost-effective devices. AR navigation of pedicle screw placement has been demonstrated to be feasible with high precision in a self-built setup based on two phantoms of the lumbar spine [15], and AR navigation improves the precision in drilling pilot holes for pedicle screws in a laboratory setting [16]. To our knowledge, AR guidance has never been described in the context of acetabular osteotomies (OT), and therefore, its potential merits are largely unknown.

We performed a proof of concept study to find the potential of AR for the guidance of the surgeon while performing PAOs with oscillating saw and OT-chisels using an AR-HMD.

The study questions were the following:

1. Is it feasible to execute a 3D preoperative planning of a PAO with AR guidance?
2. Is there a difference in accuracy between AR-guided PAO as against the freehand (FH) technique?
3. Is there a difference between the performance of an experienced PAO surgeon and a novice both assisted by AR guidance?

## Materials and methods

**Fig. 1:**
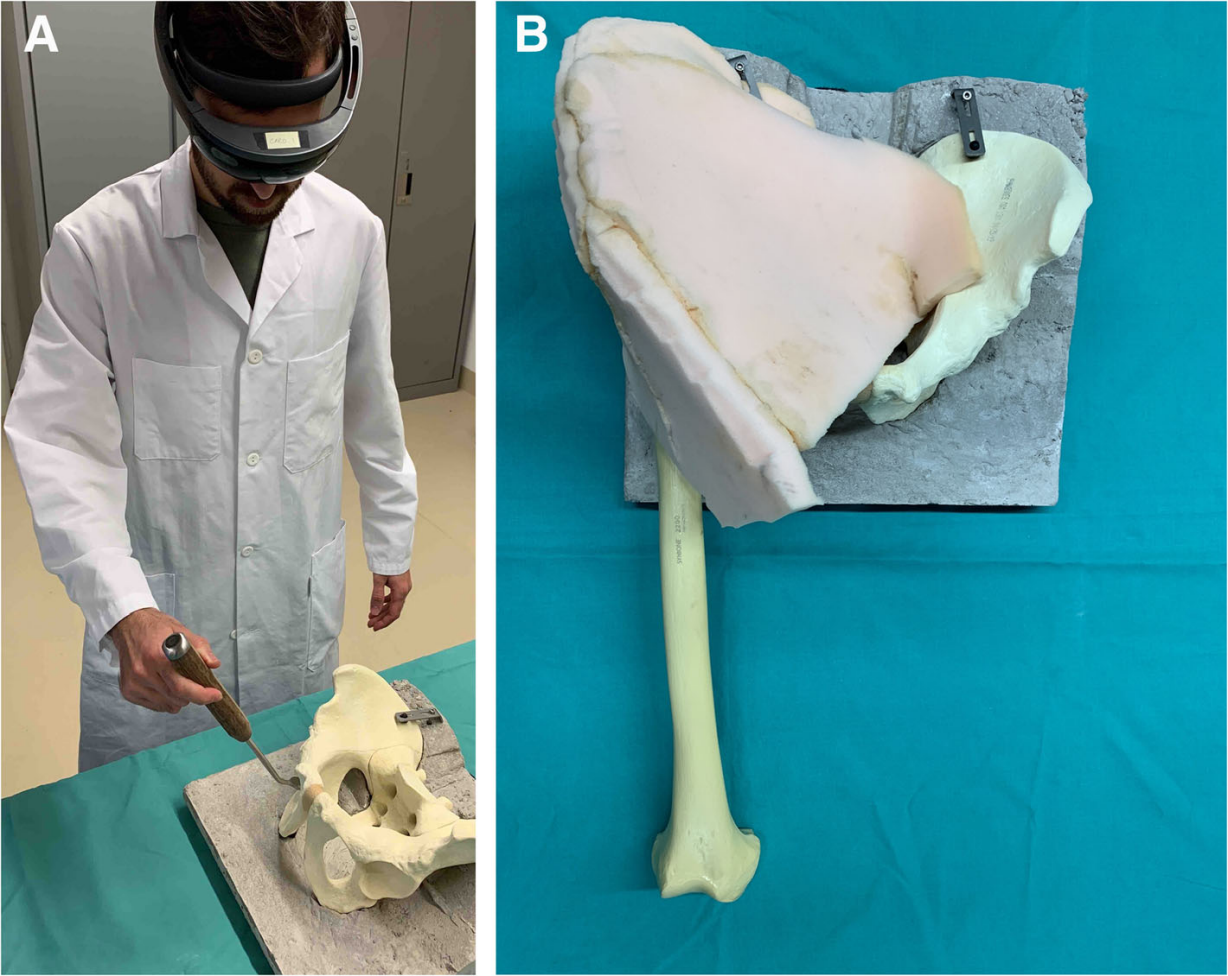
Demonstration. **a** The surgeon holds the curved chisel wearing the HoloLens, an AR-HMD. The foam cover and the femur were removed for better visualization. **b** Sawbone pelvis with the right femur with foam simulating the soft tissue and skin

Sawbone models, consisting of the femur and pelvis, manufactured by Sawbones® (Malmoe Schweden), were manually embedded in synthetic foam (Polyurethane foam, Gummi Roost AG, Schaffhausen, Switzerland) to simulate skin and soft tissue (Fig. 1a, b).

**Fig. 2:**
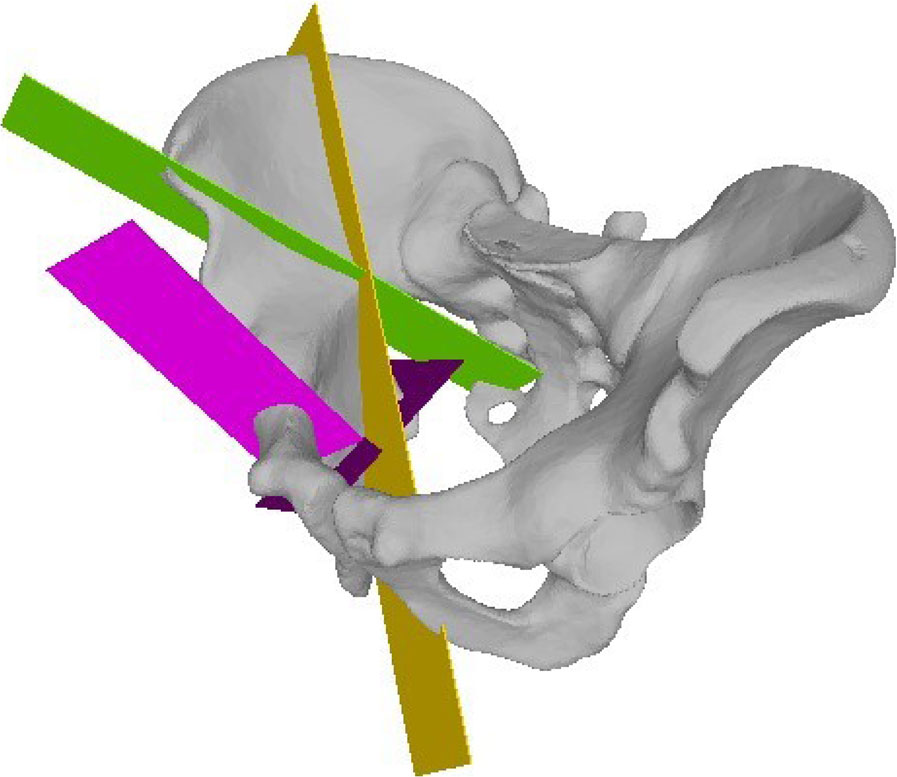
Pre-intervention planned osteotomies. All four osteotomies are depicted in this figure after the surgeon placed them appropriately in the 3D planning software

**Fig. 3:**
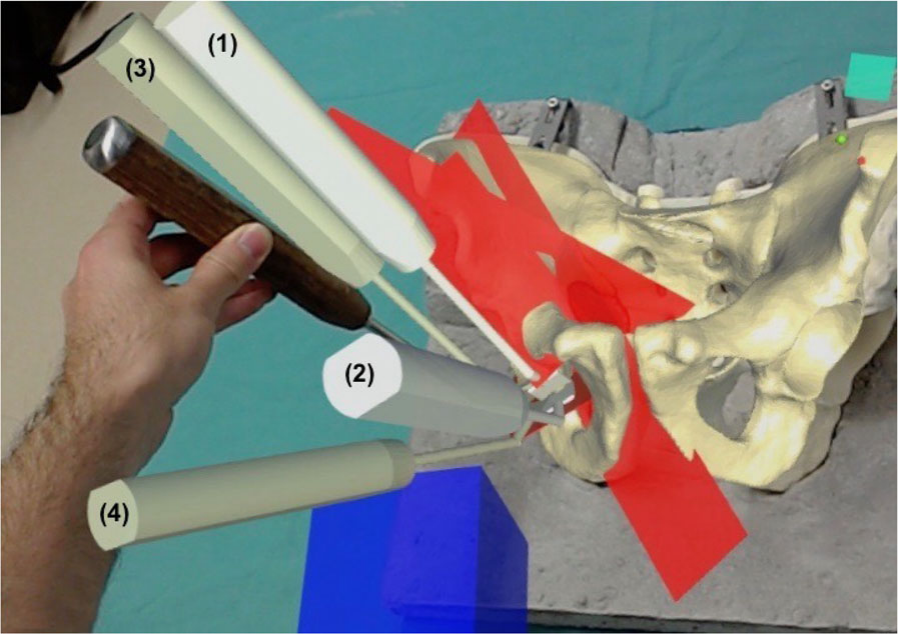
Superimposition of the 3D surgical plan of the PAO onto the sawbone pelvis. During the intervention, the surgeon can adjust the transparency of the 3D holograms to simultaneously see the chisel. Numbers 1 to 4 show the four positions of the chisel to perform the complex round ischial OT

Prior to the AR-guided procedure, a Computed Tomography (CT) scan of the sawbones was acquired with a 64-detector row scanner (Somatom Definition AS, Siemens Healthcare, Erlangen, Germany) with a tube voltage of 120 kV and a slice thickness of 1 mm. Then 3D surface models of pelvis and femur were extracted from the CT data using commercially available segmentation software (Mimics 19.0, Materialise, Loewen, Belgium). Thereafter, the 3D models were imported into the preoperative planning software CASPA (Balgrist CARD AG, Zürich, Switzerland) to preoperatively simulate the PAO osteotomies on the 3D bone models (Fig. 2). The osteotomy planes were defined by the fellowship-trained, PAO-experienced hip surgeon (POZ) according to the original description of Ganz [1] to represent supraacetabular (saOT), ischial (iOT), pubic (pOT), and retroacetabular (raOT) osteotomies. The start and end positions of each osteotomy were marked by small spheres (1-mm diameter). A 3D model of a chisel was utilized to better define the complex iOT (Fig. 3). The preoperative plan was deployed on the Microsoft HoloLens 1 (Microsoft Corporation, Redmond, WA, USA) as a custom-made, holographic Universal Windows Platform (UWP) application using Unity (version 2017.4.9f1 Personal (64bit), Unity Technologies, San Francisco, CA, USA) and Microsoft Visual Studio (version Community 2017, Microsoft Corporation, Redmond, WA, USA).

### Surgical technique

**Fig. 4:**
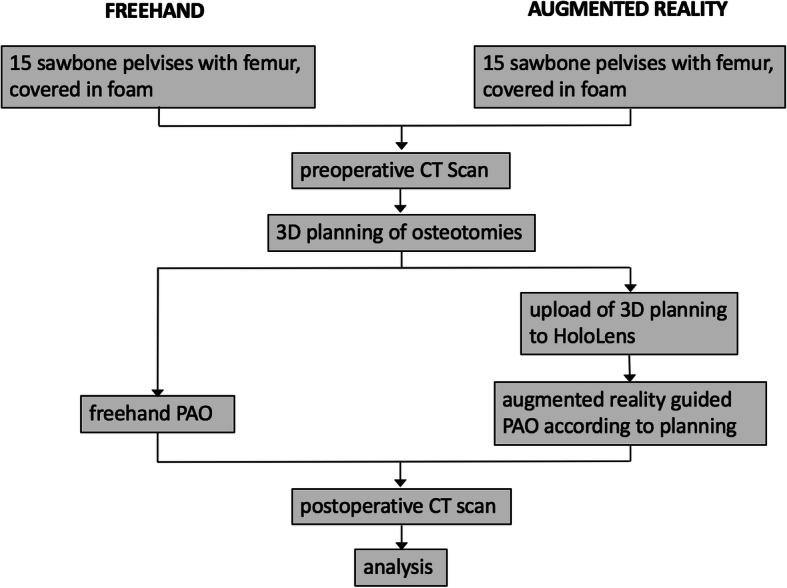
Study design. A flowchart of the study design

One hip consultant with experience on > 100 PAOs (PAO surgeon) and another hip consultant with no PAO experience (novice surgeon; assisted in > 50 PAO surgeries) performed 15 AR-guided and 15 FH PAOs each on the sawbone models according to Ganz [1] through a simulated Smith-Peterson incision of the covering foam. The two consultants performed the PAOs in the same setting in alternating order, starting with the FH. For both the surgeons, the study setup was identical (Fig. 4).

#### AR-navigated PAO

**Fig. 5:**
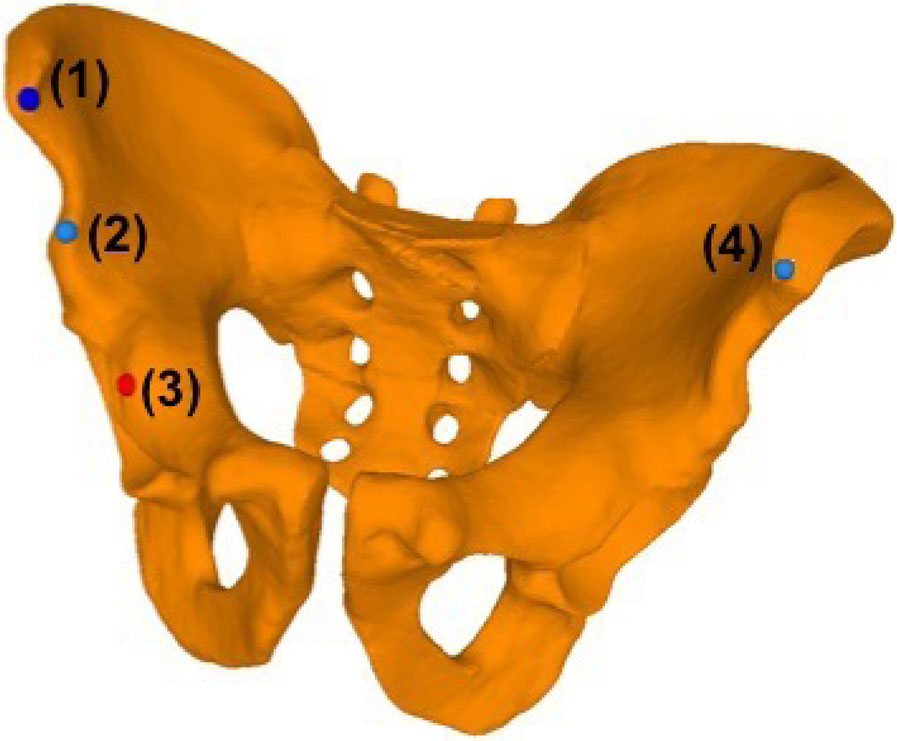
Pelvis with landmark registration points. The landmarks used for registration points were as follows: (1) right anterior superior iliac spine, (2) right anterior inferior iliac spine, (3) right eminentia iliopubica, (4) left anterior superior iliac spine

AR navigation requires registration, which means the process of creating a correspondence between pre- and intra-procedure anatomy to superimpose the 3D holograms of the osteotomy planes onto the real situs. Such registration was done by marking the 3D position of pelvic landmarks with a pointing device (Fig. 5). The technical description of the method used here for surface digitalization and registration can be found in a previous description [15]. After registration, the pelvic bone, the planned osteotomy planes, and the model of the chisel for the ischial osteotomy were displayed in situ. The visual control was exercised by the surgeon. The registration was redone if the holographic model did not exactly coincide with the sawbone model. The surgical approach was identical to the control group. The dedicated curved PAO chisel was used for the first partial ischial OT, but this time, the surgeon was guided by holographic displays of graphical models of the four positions in which the curved chisel was needed to be held to perform the desired osteotomy (Fig. 5). Thereafter, the pubic OT was performed with a straight chisel, also with AR guidance. The third supraacetabular OT was performed with an oscillating saw aligned with the displayed holographical model of the osteotomy plane. To complete the partial ischial OT, a straight chisel was used along the posterior acetabulum by displaying the holographical model of the osteotomy plane for orientation. The surgeons were instructed to perform the osteotomies exactly as they had been shown in the holographic model.

#### FH PAO

A dedicated curved PAO chisel was used for the first partial ischial OT, which (under real circumstances) has to be performed without visual control because the region is covered by the capsule and the proximal femur in the in vivo situation. Next, the pubic cut was performed with a straight chisel. The third supraacetabular OT was performed with an oscillating saw. To complete the partial ischial OT, a straight chisel was used along the posterior acetabulum aiming to achieve the desired angle of 120° until the acetabulum became mobilized.

#### Evaluation of accuracy

**Fig. 6:**
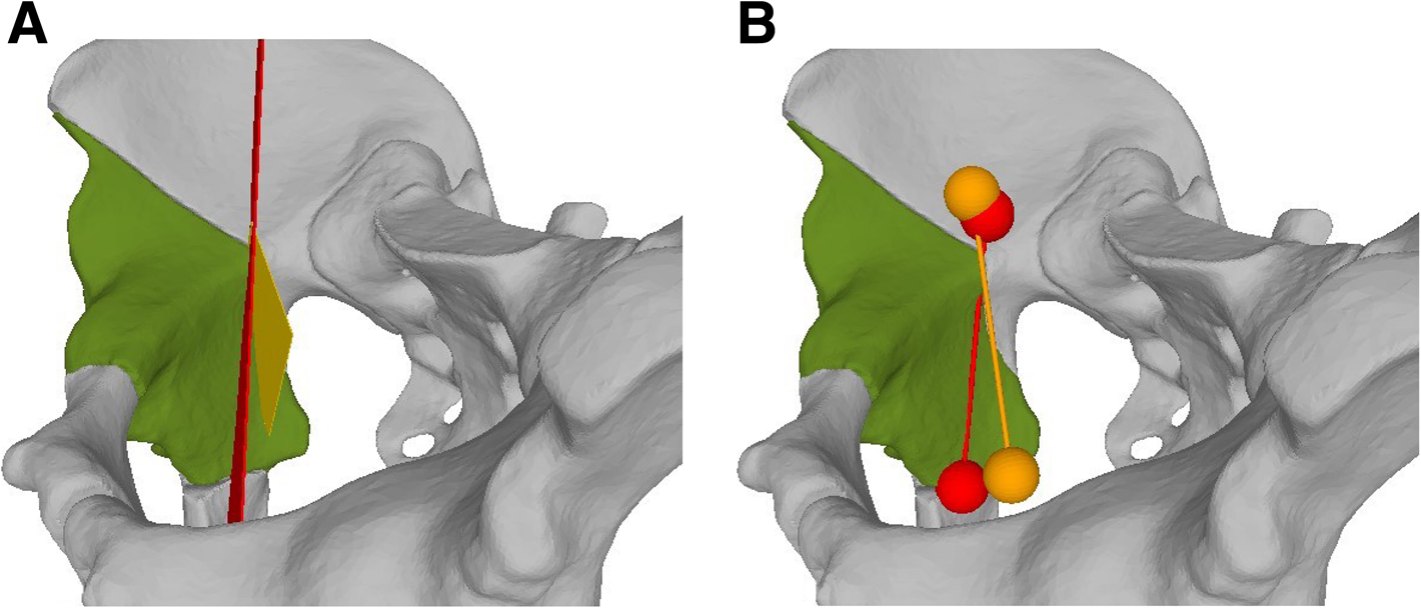
Evaluation of the retroacetabular angle. **a** Post-intervention 3D projection of the retroacetabular angle. The planned osteotomy plane of the crucial retroacetabular OT is depicted in red and the yellow plane shows the actual performed osteotomy plane. **b** 2D projection of the 3D planes. The angle between these two planes was measured in a 2D fashion. A large aberration of the performed to the planned angle could lead to a posterior column or intraarticular osteotomy

After the procedures described above were carried out, CT scans of each of the 60 pelvic sawbone models were acquired and 3D models of the residual pelvic bones were generated using the Mimics software. Each residual pelvic bone was imported into the CASPA software. The iterative closest point functionality of the software was used to superimpose the residual bone on the preoperative plan, and 3D plane objects were fitted manually to the osteotomy surfaces of the residual bone. The osteotomy starting points were defined by small spheres (1 mm diameter). The accuracy of the osteotomy entry point was then determined and evaluated by calculating the 3D difference between the sphere centers in planned and performed positions. As an error in the direction of the retroacetabular, OT can result in severe complications (intraarticular osteotomy or fracture of the posterior column). Additionally, the angular deviation between the planned and performed raOT was evaluated. For this, the OT planes were projected as lines on the medial top view of the surgical approach (Fig. 6). Subsequently, the 2D angle between the lines was measured. Lastly, potential surgical failures were assessed in terms of osteotomies penetrating the acetabulum or interrupting the posterior column.

#### Statistics

**Fig. 7:**
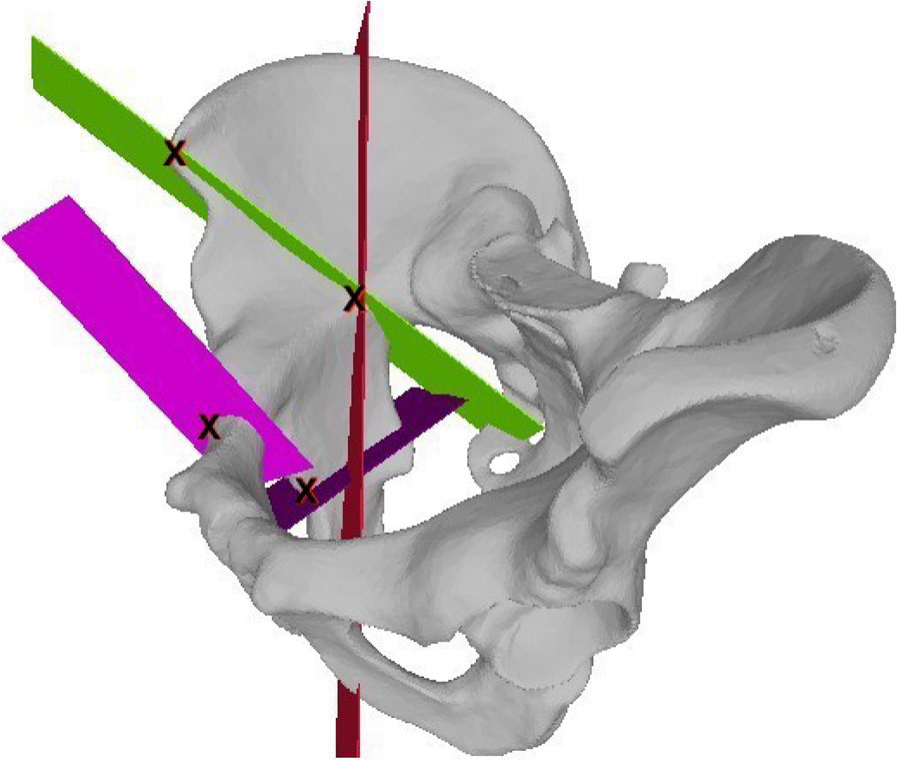
Start points of the osteotomies. The X depicts the starting points of the four osteotomies where the differences from the planned to the performed freehand and AR-guided PAO were measured

Statistical analysis was conducted using IBM SPSS Statistics v24.0 (SPSS, Chicago, IL). Descriptive statistics, one-way univariate analysis of variance, and a paired Student *t* test were used to compare the pre-intervention planned starting points and executed starting points (Fig. 7), and the 2D angle of the raOT was measured (Fig. 6). The result was considered statistically significant when *p* < 0.05.

## Results

The experienced PAO surgeon achieved the following mean differences between planned and executed starting points of the OTs: In the saOT, the difference was 1.2 mm [± 0.8] for the FH vs. 1.5 mm [± 1.0] in the AR-guided PAO. In the pOT, the difference was 2.1 mm [± 1.1] in the FH vs. 3.5 mm [± 2.3] in the AR PAO. In the iOT, the difference was 2.4 mm [± 3.1] in the FH vs. 2.7 mm [± 1.7] in the AR-guided PAO. Lastly, in the raOT, the difference was 2.0 mm [± 1.7] in the FH vs. 1.8 mm [± 1.6] in the AR-guided PAO. The differences did not reach statistical significance.

With AR navigation, the raOT angle had a significantly better correlation with the pre-intervention plan than in the FH group (7.8° [± 2.7] vs. 11.7° [± 3.5], *p* = 0.0027). No intraarticular osteotomies or interruption of the posterior column occurred in any of OTs performed by the experienced surgeon

The novice surgeon achieved the following mean distances between planned and the executed starting points: In the saOT, the difference was 2.4 mm [± 1.3] in the FH vs. 1.5 mm [± 1.6] in the AR PAO. In the pOT, the difference was 6.1 mm [± 2.7] in the FH vs. 4.5 mm [± 3.6] in the AR-guided PAO. In the iOT, the difference was 3.2 mm [± 3.0] in the FH vs. 1.3 mm [± 1.6] in the AR-guided PAO. In the raOT, the difference was 2.6 mm [± 1.5] in the FH vs. 1.8 mm [± 1.9] in the AR-guided PAO. The iOT was the only osteotomy showing a statistical difference (*p* = 0.0027). The retroacetabular angle was without significant difference between FH and AR-guided PAOs: 8.8° [± 3.7] vs. 7.2° [± 3.2], *p* = 0.554), respectively. One intraarticular osteotomy was encountered in the FH group.

Detailed information is provided in Table 1.

**Table 1:** A complete overview of the results regarding the starting points of the freehand and the AR PAO of the four osteotomies and the retroacetabular angle for both the PAO and the non-PAO surgeon

Osteotomies (PAO surgeon)	Freehand PAO [range (min., max.); ± SD] Mean distance	Augmented reality PAO [range (min., max.); ± SD] Mean distance	***p*** value
**Supraacetabular (saOT)**	1.2 mm [0.37, 2.74; ± 0.77]	1.5 mm [0.08, 3.22; ± 1.01]	0.424
**Pubis (pOT)**	2.1 mm [0.61, 4.04; ± 1.10]	3.5 mm [0.42, 6.51; ± 2.29]	0.067
**Ischium (iOT)**	2.4 mm [0.34, 12.6; ± 3.12]	2.7 mm [0.7, 7; ± 1.67]	0.762
**Retroacetabular (raOT)**	2.0 mm [0, 6.4; ± 1.65]	1.8 mm [0, 4.9; ± 1.58]	0.688
**Osteotomies (non-PAO surgeon)**	**Freehand PAO [range (min., max.); ± SD]** **Mean distance**	**Augmented reality PAO [range (min., max.); ± SD]** **Mean distance**	***p*** **value**
**Supraacetabular (saOT)**	2.4 mm [0.53, 4.74; ± 1.32]	1.5 mm [0.00, 3.77; ± 1.56]	0.523
**Pubis (pOT)**	6.1 mm [2.97, 12.02; ± 2.73]	4.5 mm [1.40, 15.05; ± 3.64]	0.544
**Ischium (iOT)**	3.2 mm [0.10, 10.25; ± 2.95]	1.3 mm [0.00, 5.36; ± 1.58]	0.030
**Retroacetabular (raOT)**	2.6 mm [0.51, 5.02; ± 1.48]	1.8 mm [0, 7.18; ± 1.92]	0.812
**Angle (PAO surgeon)**	**Freehand PAO [range (min., max.); ± SD]**	**Augmented reality PAO [range (min., max.); ± SD]**	***p*** **value**
Retroacetabular angle	11.7° [7.53, 20.55; ± 3.52]	7.8° [4.21, 14.11; ± 2.67]	0.0027
**Angle (non-PAO surgeon)**	**Freehand PAO [range (min., max.); ± SD]**	**Augmented reality PAO [range (min., max.); ± SD]**	***p*** **value**
Retroacetabular angle	8.87° [3.03, 15.75; ± 3.74]	7.23° [2.86, 13.89; ± 3.16]	0.554

## Discussion

While the PAO technique has been well-described since its introduction [1], it remains a technically demanding procedure with significant risks of complications [5, 6, 17] and a shallow learning curve [4] and having worse outcomes when overcorrecting [18]. The perfect and safe performance of the four osteotomies in PAO remains a challenge, and therefore, an easy, safe guidance would be preferable.

This proof of concept study is the first to prove the feasibility and high accuracy of AR-guided PAOs and investigate the stability of the results in the dependence of different levels of surgical experience. Therefore, the answer to the first question posed in this study is answered in the affirmative. Even though no significant differences were apparent for the OT starting points for the experienced PAO surgeon, the most difficult ischial OT, however, was significantly more accurate with AR guidance for the novice surgeon. Interestingly, the angle of the AR-guided raOT was only more precise for the PAO surgeon and not different for the novice surgeon when compared to FH. This observation can be interpreted to have statistical but not clinical relevance because a deviation of 11° from a correct starting point and along a short path does not necessarily result either in an interruption of the posterior column or an intraarticular OT. While the experienced PAO surgeon did not make a malpositioned OT reaching the posterior column or the joint, the novice surgeon, while working FH, i.e., without AR guidance, performed one intraarticular osteotomy. Additionally, the ischial OT, considered technically as the most challenging, had a significantly higher correlation with the pre-intervention plan with AR guidance compared to FH for the novice surgeon. Therefore, the second and the third study questions are not answered unequivocally in the affirmative or negative. However, the results were discussed in the section above.

Even though the proof of concept of AR-guided OTs for PAOs has now been established, further alternatives need to be discussed to decide whether further research and development of such technologies for bringing them into clinical practice is justified by their potential.

To improve the accuracy and reliability of osteotomy, several supplementary tools have been employed, including navigation systems with fluoroscopy, with infrared tracking, and with patient-specific guides [7–12]. The optical tracking guidance has the downside of requiring large expensive equipment, and other additional trackers are always needed, which necessitates additional incisions. Furthermore, the downside of this type of guidance is that the surgeon has to move his head out of the operating field to view the computer-aided guidance. The patient-specific instrumentation guidance has the advantage of being very precise but requires larger incisions because the osteotomy guides are bulky. Furthermore, these guides have to be manufactured individually, which is time-consuming and costly.

In our opinion, the guiding system with the AR technique that we tested has very great potential because of its three advantages. First, OT poses a 3D problem and AR technology facilitates making a 3D plan to solve it and execute it in 3D, taking the joint loading forces into account. Second, the guidance is directly in the surgeon’s field of vision and does not need the surgeon to take his sight away from the incision. Third, due to the overlay of the osteotomies and also the guidance of the chisel, it is possible to guide the most important osteotomy in PAO, namely, the first ischial partial osteotomy.

Therefore, we believe that further research and development of AR-guided OTs are needed to capitalize on its potential merits as a cost-effective and more intuitive navigation method.

This proof of concept study does not claim for the direct application of the technology in clinical care because the limitation of the presented setup needs consideration: Certainly, the surgical setup with the sawbone pelvis/femur models covered with foam does not resemble the in vivo situation. Therefore, the next step is the execution of cadaver studies. Additionally, in this study, only the osteotomies were AR-guided, and the feasibility of navigating the reorientation and fixation of the acetabulum in a new, corrected position was not investigated. However, this study could be a new platform to establish other merits of AR guidance. As the surgeons performed the AR-guided and FH PAOs alternately and in one setting, there probably was a memory effect, especially for the FH group. But despite the memory effect that may have helped in the FH group, the osteotomies in the AR group were more reliable.

## Conclusion

This is the first proof of concept study documenting the feasibility and reporting on the accuracy of AR-guided osteotomies in PAO. Our study showed that the holographic surgical navigation of the PAO surgery is feasible and precise in a realistic pelvis-hip model using the Hololens AR headset. The study shows that AR-guided OTs are safe to perform, showed a high correlation with the preoperative plan, and were as precise as osteotomies performed in an FH fashion by an experienced PAO surgeon.

## Data Availability

All data that were generated or analyzed during this study are included in this published article.

## Abbreviations

PAO: Ganz’ periacetabular osteotomy
OT: Osteotomy
saOT: Supraacetabular osteotomy
pOT: Pubic osteotomy
iOT: Ischial osteotomy
raOT: Retroacetabular osteotomy
AR: Augmented reality
FH: Freehand
PPM: Anatomic plastic pelvis model
IMU: Inertial measurement units
HMD: Head-mounted displays
UWP: Universal Windows Platform
